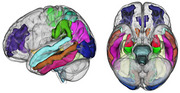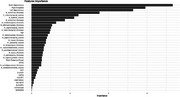# Multimodal approach to diagnose Alzheimer's dementia using risk factors and MR imaging features derived from mild‐moderate AD patients

**DOI:** 10.1002/alz70856_097694

**Published:** 2025-12-24

**Authors:** Geetha Chilla

**Affiliations:** ^1^ Bioinformatics Institute, A*STAR, Singapore, Singapore, Singapore

## Abstract

**Background:**

Increase in AD specific neuropathological burden (ADNPC) leads to appearance and progression of clinical symptoms (Jack et al., 2024), suggesting that in‐vivo markers could capture earliest symptomatic presentations in AD. In this regard, clinical and neuroimaging data from mild‐moderate AD patients were analyzed in an effort to identify AD at earlier clinical stages. A multimodal diagnostic framework was built using risk factors and MRI brain markers and evaluated using random forests classification.

**Method:**

OASIS‐3 database, consisting of longitudinal data of 800+ individuals who are cognitively normal/at various stages of cognitive decline (CDR score: 0‐2) was employed for this study. Data was split into healthy controls, transition group and 3 severity groups of AD patients based on clinical diagnosis & atrophy levels. Using Freesurfer, generalized linear mixed models & Bayesian methods, causal MR features discerning mild‐moderate AD with healthy ageing were extracted. Separately, non‐imaging risk factors associated with AD also were extracted from among demographics data (Age, APOE), family history of dementia (parents, siblings, kids & relatives) and subject medical history such as presence of cardiovascular diseases, TBI, stroke, depression, if underwent medical procedures etc. Identified MR features and risk factors were then used to build a random forest classification model to evaluate their effectiveness in diagnosing AD.

**Result:**

Causal relationships were found between mild‐moderate AD and 24 regions of brain including Hippocampus, entorhinal cortex, inferior temporal and parahippocampal gyrus. Identified AD risk factors, in addition to age, include being an APOE ɛ4 carrier, maternal history of dementia, and presence of conditions such as hypertension, depression, stroke, transient ischemic attack, cardiac arrest, B12 deficiency, urinary & bowel incontinence. With these 35 features and random forest modelling on a majority class under‐sampled dataset, a balanced accuracy of 92% with sensitivity & specificity of 97% & 87% were achieved. On a subset of data with at least one missing clinical factor, accuracy, sensitivity & specificity were 66%, 61% & 71%.

**Conclusion:**

Integrating specific MRI features and risk factors could help in detecting at‐risk and early AD patients.

This work was funded by the AD Data Initiative (ADDI) and was carried out on AD Workbench (2020).